# Combined Transcutaneous Spinal Stimulation and Locomotor Training to Improve Walking Function and Reduce Spasticity in Subacute Spinal Cord Injury: A Randomized Study of Clinical Feasibility and Efficacy

**DOI:** 10.3390/jcm10061167

**Published:** 2021-03-11

**Authors:** Stephen Estes, Anastasia Zarkou, Jasmine M. Hope, Cazmon Suri, Edelle C. Field-Fote

**Affiliations:** 1 Shepherd Center, Crawford Research Institute, Atlanta, GA 30309, USA; stephenpestes@gmail.com (S.E.); anastasia.zarkou@shepherd.org (A.Z.); jasmine.hope@shepherd.org (J.M.H.); cazmon.suri@shepherd.org (C.S.); 2 Graduate Division of Biological and Biomedical Sciences, Laney Graduate School, Emory University, Atlanta, GA 30322, USA; 3 Division of Physical Therapy, Emory University School of Medicine, Atlanta, GA 30322, USA; 4 Program in Biomedical Sciences, Georgia Institute of Technology, Atlanta, GA 30332, USA

**Keywords:** activity-based therapy, gait, locomotion, neuromodulation, paraplegia, task-specific training, tetraplegia, use-dependent plasticity

## Abstract

Locomotor training (LT) is intended to improve walking function and can also reduce spasticity in motor-incomplete spinal cord injury (MISCI). Transcutaneous spinal stimulation (TSS) also influences these outcomes. We assessed feasibility and preliminary efficacy of combined LT + TSS during inpatient rehabilitation in a randomized, sham-controlled, pragmatic study. Eighteen individuals with subacute MISCI (2–6 months post-SCI) were enrolled and randomly assigned to the LT + TSS or the LT + TSS_sham_ intervention group. Participants completed a 4-week program consisting of a 2-week wash-in period (LT only) then a 2-week intervention period (LT + TSS or LT + TSS_sham_). Before and after each 2-week period, walking (10 m walk test, 2-min walk test, step length asymmetry) and spasticity (pendulum test, clonus drop test, modified spinal cord injury—spasticity evaluation tool) were assessed. Sixteen participants completed the study. Both groups improved in walking speed and distance. While there were no significant between-groups differences, the LT + TSS group had significant improvements in walking outcomes following the intervention period; conversely, improvements in the LT + TSS_sham_ group were not significant. Neither group had significant changes in spasticity, and the large amount of variability in spasticity may have obscured ability to observe change in these measures. TSS is a feasible adjunct to LT in the subacute stage of SCI and may have potential to augment training-related improvements in walking outcomes.

## 1. Introduction

The loss of motor control is a hallmark of spinal cord injury (SCI), impacting the health and quality of life for persons with a SCI. To overcome these impairments, rehabilitation therapies aim to improve motor control and maximize functional recovery by optimally activating spared pathways. For walking function, rehabilitation therapy focuses on locomotor training (LT), which involves repetitive stepping with or without manual or robotic assistance, often augmented by body-weight support in either the treadmill or overground environments [1]. Through repetition, LT is thought to activate use-dependent neural mechanisms within spared neuronal circuits to improve motor control and walking function [2]. While improvements in walking function have been observed following LT, randomized studies have demonstrated that no single approach appears to be superior to others [3]. Overall, improvements have been modest and it has been suggested that combination approaches may offer the greatest promise. In recent years, there has been increased interest in combining traditional LT strategies with novel neurotherapeutic approaches to amplify these mechanisms [4,5,6]. 

Spinal stimulation is a neuromodulation strategy that has the potential to amplify the inputs of therapies like LT that are intended to promote use-dependent neuroplasticity and improve walking outcomes. Case reports and series on persons with chronic motor-complete and severe incomplete SCI have shown that intensive LT alone was insufficient for improving volitional motor control and walking function; however, with the addition of surgically implanted epidural spinal stimulators, these same individuals showed improvements in volitional muscle activation and walking function [7,8,9]. 

While often referred to as “spinal cord stimulation”, evidence has shown that the effects of epidural stimulation are mainly attributable to activation of peripheral nerve roots, primarily the large-diameter afferent fibers. The same spinal circuits that are engaged by epidural stimulation are also engaged via transcutaneous spinal stimulation (TSS), which uses electrodes placed on the skin over the vertebrae [10,11]. Definitive evidence for the mechanism whereby spinal stimulation facilitates volitional movement in persons with SCI is not available; however, a reasonable theory is that summation of the weak, subthreshold descending signals related to volitional effort and the stimulation-induced increase in excitability of spinal circuits together are sufficient to bring the motoneurons to threshold. Therefore, combining LT and TSS may represent a non-invasive and clinically accessible approach to improving walking function. While proof-of-concept studies have suggested that the combination of TSS and LT appears to have value for improving walking speed [12] and step kinematics [5], the lack of a control group in these studies makes it difficult to determine whether these effects were due to the addition of TSS or simply to LT.

For many persons with SCI, the loss of volitional muscle activation accounts for only part of their motor control impairments. Upwards of 78% of persons with a SCI experience spasticity [13], characterized by sustained or intermittent involuntary muscle activity that manifests as spasms and stiffness [14]. While the impact of spasticity can vary from person to person, it has been shown to have an overall negative impact on an individual’s quality of life and can interfere with daily activities such as sleep and mobility [15,16,17]. Spasticity can hinder rehabilitative efforts, and lead to a range of gait abnormalities that impact walking [16]. In those who experience problematic spasticity, its management is a top priority. Oral antispasmodic medications are often used as the first line of treatment for managing spasticity; however, other than for tizanidine, evidence for their effectiveness is lacking, and they carry unwanted side effects such as drowsiness, muscle weakness, and lethargy [18,19]. People with SCI report that movement-related activities are among the strategies that they find most useful for managing their spasticity [20], and studies have shown that LT is associated with reductions in spasticity [21,22,23]. Likewise, there is evidence that spinal stimulation may have value for the management of spasticity in persons with SCI [24,25,26,27,28].

Since both LT and TSS each have the potential to improve walking function and reduce spasticity, it seems reasonable that using these approaches concurrently could be associated with larger effects. Since one of the advantages of TSS is that it is a clinically accessible approach, the purpose of this study was to assess the feasibility and preliminary efficacy of combining LT with TSS as a component of real-world clinical rehabilitation. Using a randomized, sham-controlled, pragmatic study design, we aimed to assess the effects of TSS when used as an adjuvant to 4 weeks of therapist-directed, usual care LT. While this design deviates from the standard clinical explanatory study design and increases variability based on the variety of LT approaches the clinician can use, this pragmatic design more closely mirrors real-world clinical practice and can therefore improve the translatability of study findings to the clinical setting. 

## 2. Materials and Methods

This study was carried out with the ethics approval from the Shepherd Center Research Review Committee (Project #696). All participants provided written informed consent prior to study enrollment in accordance with the Declaration of Helsinki, and the study was conducted in accordance with the Health Insurance Portability and Accountability Act (HIPAA) guidelines. This study was registered with clinicaltrials.gov (NCT03240601).

### 2.1. Subjects

We captured data related to both feasibility of applying TSS as part of a program of usual care LT, as well as data related to the impact of LT + TSS on walking and spasticity measures. Participants were recruited from the inpatient clinical services of a specialty rehabilitation hospital. Recruitment was via advertisements, information provided in a monthly research education class, and information conveyed to potential participants by clinical staff. To be eligible for the study, participants had to be in the subacute rehabilitation phase of the SCI (2–6 months post-injury) and had to qualify for participation in a clinical LT program as determined by their physical therapist. Individuals were eligible for participation if they met the following inclusion criteria: 16–65 years of age with SCI, ability to take at least one step with or without an assistive device, and presence of at least mild spasticity affecting the lower extremity muscles (as determined by participant self-report). Individuals with the following exclusion criteria were not considered for participation: neurological level of injury at or below T12, progressive or potentially progressive spinal lesions (including degenerative or progressive vascular disorders of the spine and/or spinal cord), history of cardiovascular irregularities, difficulty following instructions, orthopedic problems that would prevent participation in study interventions (i.e., knee or hip flexion contractures > 10 degrees), women who were pregnant, or had reason to believe may become pregnant, persons who have implanted stimulators/electronic devices of any type, and persons with an active infection of any type.

### 2.2. Study Design

**Figure 1:**
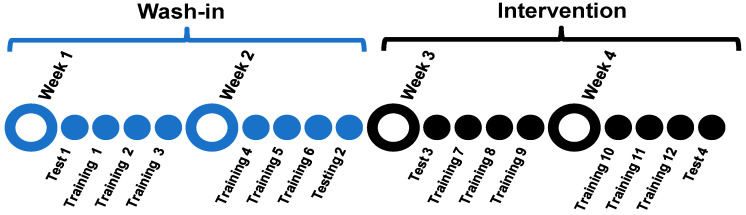
Study design.

We used a wash-in, randomized, control study design (Figure 1) consisting of four consecutive weeks of LT directed by physical therapists. For the first two weeks, participants received 6 bouts of LT. For the last 2 weeks, participants were randomized to receive 6 bouts of either 30 min of TSS coupled with LT or a sham-control stimulation coupled with LT. Primary outcome assessments for walking function and spasticity were conducted at the beginning and end of each 2-week intervention block (first 2 weeks (wash-in phase) and second 2 weeks (intervention phase)). The assessments at the end of each 2-week block were performed prior to the training on that day. Pre- and post-training assessments of spasticity and assessments of tolerability (see below) were performed on each training day. 

### 2.3. Intervention

Intervention: LT approaches used in the study included treadmill-based training with or without body weight support and with or without manual or robotic assistance (Lokomat, Hocoma, Volketswil, Switzerland), as well as overground locomotor training with or without body weight support and with or without manual assistance. LT approaches for each participant were chosen by the treating therapist in accordance with standard clinical practice. LT duration was approximately 1 h and included both setup and take down time of all equipment.

Transcutaneous spinal cord stimulation (TSS): Electrical stimulation (50 Hz, biphasic pulses) was applied using a portable electrotherapy device (Empi Continuum, DJO Global, Vista, CA, USA), as previously described [28]. Briefly, with the participant seated, the stimulating electrode (5 cm round electrode) was placed over vertebral levels T11/T12, identified via manual palpation, and the reference electrode (10 cm × 15 cm butterfly electrode) was placed over the umbilicus. With the participant standing, the stimulator was turned on and adjusted to a level at which the participant reported sensations of tingling in the legs and feet or to the highest level the participant could tolerate. For those with impaired sensation as well as those with sensation, target stimulation was subthreshold for lower extremity muscle activation and verified by the absence of visible muscle contraction. Stimulation was delivered for 30 min at the target stimulation intensity and occurred concomitantly with LT. Following 30 min of stimulation, the intensity was ramped down and the stimulation unit was turned off and disconnected. If LT finished prior to the 30 min of stimulation, then participants completed their 30 min of stimulation while seated on a high-lo mat where training day assessments would occur. Conversely, if the 30 min of stimulation finished prior to the end of LT, then the LT session was completed prior to the assessments. 

Sham-control stimulation—TSS_sham_: The sham-control was designed to control for placebo effects associated with the perception of intervention. For the TSS_sham_, a 2-inch round electrode was placed over T11/T12 and a reference electrode was placed over the umbilicus, as performed with TSS. The intensity of electrical stimulation was briefly ramped up to a level at which the participants reported perceiving the stimulation, then ramped down and turned off for the remainder of the intervention. The stimulator and electrodes remained attached to the participant for 30 min of the LT session, similar to the active TSS intervention. After 30 min, the stimulator unit was disconnected from the electrode leads.

### 2.4. Outcome Measures

#### 2.4.1. Walking Assessments

All walking-related data were captured during overground walking: participants were given the standardized instruction “walk as quickly and safely as possible”. Participants wore a gait belt during walking assessments and were guarded by a physical therapist for safety. During testing, participants used the walking assistive and orthotic devices they typically used during therapy, however no body weight support was provided during testing. 

10-Meter Walk Test (10MWT): We utilized the 10MWT, which was our primary outcome measure for walking speed to assess changes in walking speed at the beginning and end of each 2-week LT block. A stopwatch was used to capture walking speed. Participants completed 3 walks in each assessment session with a 2 min rest between trials.

Additionally, spatiotemporal gait characteristics were recorded during the 10MWT using a 6 m instrumented walkway (GAITRite, CIR Systems, Franklin, NJ, USA) positioned in the middle of the 10 m walking path. Each walk consisted of at least 4 consecutive footfalls over the mat. Footfall data generated by the GAITRite software were used to compute step asymmetry between the stronger and weaker leg as follows: Step length asymmetry: larger step length/(larger step length + smaller step length). Step length asymmetry was averaged across trials and used for analysis. 

2-Minute Walk Test (2MWT) was used to assess changes in walking distance at the beginning and end of each 2-week LT block. Distance was measured with a measuring wheel. 

#### 2.4.2. Spasticity Assessments

Pendulum test: The pendulum test, our primary measure of spasticity, was used to assess stretch-induced quadriceps reflex excitability based on first swing excursion (FSE) angle (recorded using motion capture software), wherein a larger angle indicates a greater excursion of the limb before the onset of reflex muscle contraction, and therefore less spasticity [29]. Briefly, participants were positioned reclined at the edge of an adjustable height mat with both legs flexed at the knee, and with the lower leg hanging over the mat with shoes removed. Wireless sensors were strapped to both lower extremities to capture knee joint angles using inertial motion capture software (XSENS MVN, Xsens Technologies BV, Enschede, The Netherlands). Calibrated angles were verified with the leg in full extension prior to starting the test; each leg was extended and dropped separately. Pendulum responses in each leg were tested 3 times. For each leg, the average FSE in the 3 trials of each test session was used for analysis. To confirm stretch-induced quadriceps activation during the pendulum test, electromyographic (EMG) data was concurrently monitored via electrodes (Motion Lab Systems, Baton Rogue, LA) placed over the rectus femoris muscle. EMG data were monitored using Spike software (Cambridge Electronic Design Limited, Cambridge, England). Knee angle data were analyzed off-line using customized MATLAB software (MATLAB, Mathworks, Natick, MA, USA).

Ankle clonus drop test (drop test): The drop test, the plantar flexor analog of the pendulum test, was used to assess stretch-induced reflex excitability in the ankle plantar flexors. The number of clonic oscillations captured using the drop test correlates with both electrophysiologic and clinical measures of spasticity [30]. Participants sat upright with back support on the edge of a mat table, with shoes removed and socks left on. Wireless sensors were strapped to both lower extremities to capture ankle joint angles using inertial motion capture software (XSENS MVN, Xsens Technologies BV, Enschede, The Netherlands). The ball (metatarsal heads) of one foot was positioned on the edge of a platform (10 cm height). The mat height was adjusted to ensure that the hip, knee, and ankle joints were at 90-degree angles. The participants’ leg was lifted from beneath the knee until it came into contact with a T-bar positioned 10 cm above the resting position of the knee. The examiner quickly released the leg allowing the forefoot to impact the edge of the platform, eliciting a quick stretch of the plantar flexors. Responses in each ankle were tested 3 times. For each leg, the number of clonic oscillations in each trial was counted off-line, and the average number of oscillations for the 3 trials of each test session was used for analysis. Soleus EMG data were concurrently monitored using Spike software (Cambridge Electronic Design Limited, Cambridge, England), and testing resumed only when the muscle had been quiet for 30 s. Ankle joint oscillations were analyzed off-line using customized MATLAB software (MATLAB, Mathworks, Natick, MA, USA).

Modified spinal cord injury—spasticity evaluation tool (mSCI-SET): The mSCI-SET is a self-report measure of the effect of spasticity on 33 aspects of life. Scores range from −2 to +1, where negative scores indicate problematic effects of spasticity, while positive scores indicate helpful effects of spasticity [31].

#### 2.4.3. Pre/Post-Training Assessments

Spinal Cord Assessment Tool for Spastic Reflexes (SCATS): The SCATS was used as a clinical measure of spasticity that has been shown to be reliable and valid in persons with SCI [32]. The SCATS was performed on each leg immediately before and after every intervention session by masked clinical assessors. Assessors rated clonus, flexor spasms, and extensor spasms on a 4-point scale for each lower extremity. For clonus and extensor spasms, the scale was as follows: 0—no reaction, 1—mild, lasting < 3 s, 2—moderate, lasting 3–10 s, and 3—severe, lasting > 10 s. The scale for flexor spasms was determined by measuring degrees of flexion at the knee and hip on the following scale: 0—no reaction, 1—mild, <10 degrees, 2—moderate, 10–30 degrees, and 3—severe, >30 degrees. 

Tolerability of stimulation: During the 2-week intervention period, participants were asked to rate the tolerability of their stimulation (LT + TSS and LT + TSS_sham_). Using a numeric rating scale (NRS) from 0 to 10, participants were asked how tolerable the stimulation was in regard to pain, with 0 being no pain and 10 being the worst pain imaginable. Responses were taken prior to the start of the stimulation to determine baseline tolerability, and then at 1 and 30 min during stimulation. 

### 2.5. Data Analysis

Data were analyzed using SPSS (version 26; SPSS Inc., Chicago, IL, USA). Numerical and ordinal data were presented as the mean (standard error (SE)) and median (interquartile range (IQR)), respectively. For spasticity measures, data in the more- and less-impaired legs (as determined by lower extremity motor score (LEMS)), were analyzed separately. Due to our small sample size, nonparametric tests were performed [33]. Mann–Whitney U tests were conducted to examine differences between the LT + TSS and LT + TSS_sham_ groups in change in walking ability (speed, distance, step length asymmetry) and spasticity (pendulum test, clonus drop test, mSCI-SET) during the wash-in (LT only) period, and during the intervention (LT + TSS, LT + TSS_sham_) period. The Wilcoxon signed-ranks test was used to examine changes within each group during the 2 study periods. Significance was set at α = 0.05 for all analyses. We did not adjust the significance level for multiple comparisons as our measures cannot be assumed to be independent [34], and because such adjustments are known to increase the likelihood of false negatives (Type II errors) [34,35,36,37], which is problematic in preliminary studies. For within-group comparisons, we computed the effect sizes as these are considered more meaningful than *p*-values for clinical interpretation of results [35,38,39]. We computed the effect size using Hedges’ *g*, as this approach is recommended for small samples [38]. Effect sizes were interpreted per recommendations for rehabilitation studies as small effects: 0.08 to 0.15, medium effects: 0.19 to 0.36, and large effects: 0.41 to 0.67 [40].

## 3. Results

### 3.1. Feasibility

**Figure 2:**
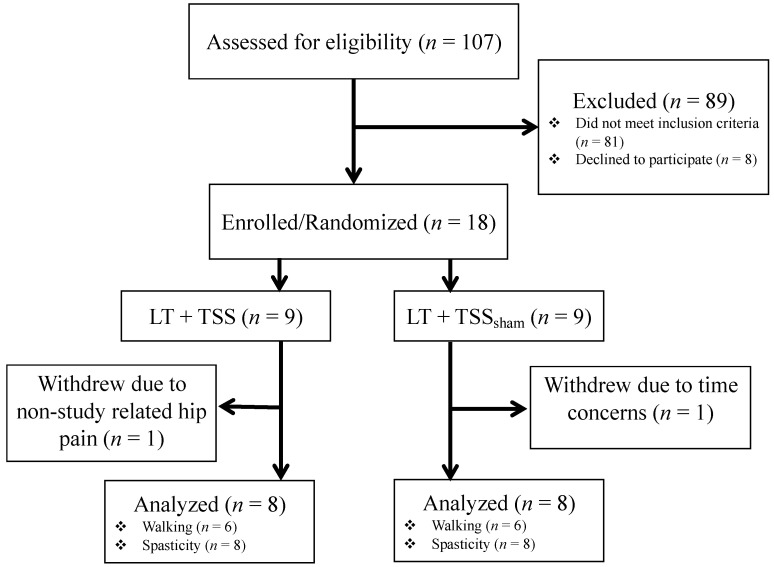
Flow diagram.

A total of 107 potential participants were screened for eligibility, and 18 were enrolled, resulting in an enrollment rate of 17% (See Flow Diagram, Figure 2). Of the 18 enrolled participants, 16 completed the study. Two participants were withdrawn after enrollment. One participant was withdrawn before training activities began because they decided to focus their time on therapy rather than study-related assessments. Another participant had developed hip pain from an event unrelated to the study, limiting mobility and thus potentially affecting study outcomes. Demographic information is presented in Table 1. No study-related adverse events were experienced by any of the participants in the study. There were no difficulties encountered with incorporating TSS into the clinical LT program. The stimulator used for the study was selected because of its availability for clinical use. However, over the course of the study, the manufacturer discontinued the production of this device. 

Of the 16 participants who completed the study (LT + TSS, *n* = 8; LT + TSS_sham_, *n* = 8), walking data was collected only from 12 participants because 4 individuals met the inclusion criteria of having the ability to take a single step, but were unable to complete the walking tests. Eleven participants completed all 12 training sessions, and 5 completed 11 sessions. Specifically, 2 participants missed a training session during the wash-in phase and 3 participants missed a single session during the intervention phase (LT + TSS group: *n* = 1; LT + TSS_sham_ group: *n* = 2). The length of each session ranged from 11 to 38 min. The mean time spent walking during LT did not significantly differ between wash-in and intervention for either group (LT + TSS group: wash-in mean time walking 27 (6) min, intervention mean time walking 29 (2) min; LT + TSS_sham_ group: wash-in mean time walking 27 (5) min, intervention mean time walking 25 (5) min). In the LT + TSS group, stimulation intensities over the course of the intervention phase were 67.60 ± 7.35 (mean ± SE, per phase of the biphasic stimulation pulse), with a range of 39 to 100 mA. Of the 8 participants in the LT + TSS group, 4 were able to tolerate stimulation intensities that induced tingling sensations in the lower extremity dermatomes, and in 4 participants, stimulation intensity at the highest tolerable level did not induce tingling sensations. In terms of stimulation tolerability, median NRS pain scores increased from 0.00 (0.09) at baseline to 3.71 (0.35) after the first minute of stimulation application, and 3.88 (0.77) at the end of stimulation application for the LT + TSS group. For the LT + TSS_sham_ group, the NRS pain scores remained the same throughout the training sessions (baseline: 0.69 (0.50), first min of stimulation: 0.69 (0.41), end of stimulation: 0.69 (0.50)). There were no participants who withdrew from the study due to stimulation-related pain.

### 3.2. Effects of TSS on Walking Ability

**Figure 3:**
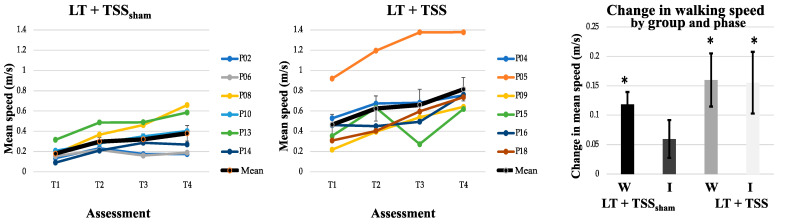
Walking speed. The mean walking speed (bold black line) increased in both the LT + TSS_sham_ (**left**) and LT + TSS (**center**) groups over time. Note that the colored lines represent the mean walking speeds of individual participants. The change in mean walking speed, calculated from the start and end of each 2-week LT phase (T1 to T2, T3 to T4; **right**) showed improvements in both groups; however, only the LT + TSS group showed continued significant improvements during the 2-week TSS intervention phase. W: Wash-in; I: Intervention. Error bars represent standard errors; * *p* < 0.05.

**Figure 4:**
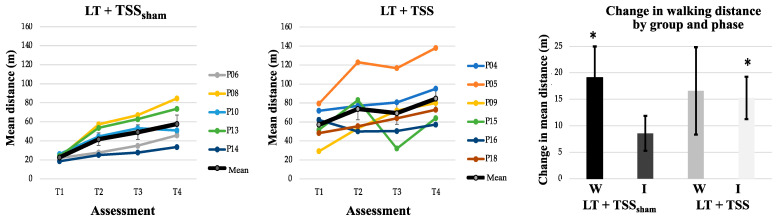
Walking distance. The average walking distance (bold black line) increased in both the LT + TSS_sham_ (**left**) and LT + TSS (**center**) groups over time. Note that the colored lines represent the average walking distance of individual participants. The change in mean walking distance calculated from the start and end of each 2-week LT bout (T1 to T2, T3 to T4; **right**) showed significant improvements in the LT + TSS_sham_ group during the wash-in phase; whereas the LT + TSS group showed significant improvements during the intervention phase. W: Wash-in; I: Intervention. Error bars represent standard errors; * *p* < 0.05.

**Figure 5:**
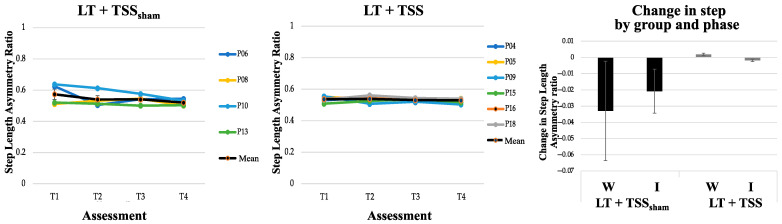
Step length asymmetry. Mean step length asymmetry (bold black line) remained the same in both the LT + TSS_sham_ (**left**) and LT + TSS (**center**) groups over time. Colored lines represent the mean step length asymmetry of individual participants. Change in mean step length asymmetry calculated from the start and end of each 2-week LT bout (T1 to T2, T3 to T4; **right**) showed only a small improvement in the LT + TSS_sham_ group. W: Wash-in; I: Intervention. Error bars represent standard errors.

Outcomes related to walking and spasticity measures are presented in Table 2. Walking speed and step length asymmetry were similar between the LT + TSS and LT + TSS_sham_ groups at baseline. The distance that participants covered during the 2MWT at baseline was significantly longer in the LT + TSS group compared to the LT + TSS_sham_ group (Z = −2.74, *p* = 0.004). The change in walking speed (*n* = 12), walking distance (*n* = 11), and step symmetry (*n* = 10) are illustrated in Figure 3, Figure 4 and Figure 5, respectively. Between-group comparisons indicated that there were no differences in change between the LT + TSS and LT + TSS_sham_ groups following the wash-in phase (walking speed: U = 13, *p* = 0.42; walking distance: U = 14, *p* = 0.86; step length asymmetry: U = 8, *p* = 0.39; pendulum (more impaired): U = 25, *p* = 0.46; pendulum (less impaired): U = 27, *p* = 0.60; ankle clonus (more impaired): U = 27.5, *p* = 0.64; ankle clonus (less impaired): U = 18, *p* = 0.14) or following the intervention phase (walking speed: U = 8, *p* = 0.11; walking distance: U = 10, *p* = 0.20; step length asymmetry: U = 8, *p* = 0.39; pendulum (more impaired): U = 22, *p* = 0.29; pendulum (less impaired): U = 30, *p* = 0.83; ankle clonus (more impaired): U = 31, *p* = 0.92; ankle clonus (less impaired): U = 25, *p* = 0.46).

Within-group analyses of walking speed (Table 2) indicated that during the wash-in phase, there were significant changes in walking speed for each of the groups (LT + TSS group: Z = −1.99, *p* = 0.05; LT + TSS_sham_ group: Z = −2.20, *p* = 0.03), with both groups exhibiting large effect sizes (*g* = 0.53 and 1.15, for the LT + TSS and LT + TSS_sham_ groups, respectively). However, during the intervention phase, only the LT + TSS group showed a significant change in walking speed (Z = −2.20, *p* = 0.03), with a large effect size (*g* = 0.43). Within-group analyses of walking distance showed that during the wash-in phase, walking distance significantly improved only for the LT + TSS_sham_ group (Z = −2.02, *p* = 0.04), however, both groups exhibited large effect sizes (*g* = 0.66 and 1.35 for the LT + TSS and LT + TSS_sham_ groups, respectively). During the intervention phase, only the LT + TSS group showed a significant change in walking distance (Z = −2.20, *p* = 0.03), with a large effect size (*g* = 0.48). Within-group analyses of step symmetry revealed that this measure did not significantly change for either of the groups during the wash-in phase, or the intervention phase, however in the LT + TSS_sham_ group, large effect sizes were observed during both the wash-in and intervention phase (*g* = 0.49 and 0.68, respectively). 

### 3.3. Effects of TSS on Spasticity

**Figure 6:**
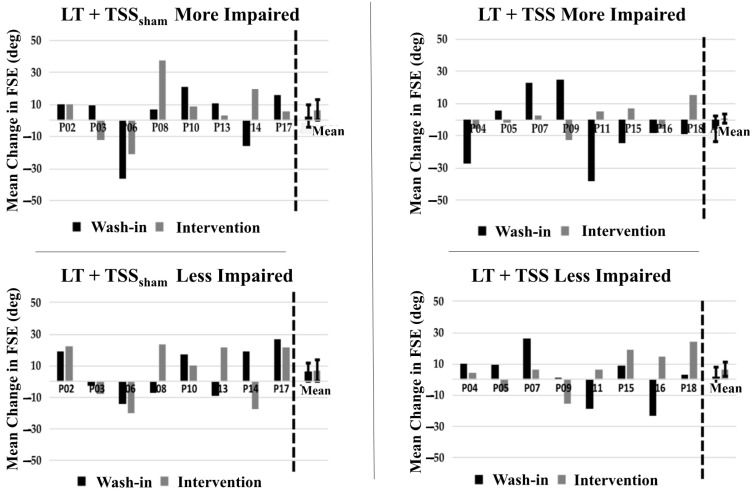
Pendulum test outcomes. No significant change in mean first swing excursion (FSE) was observed in participants in the LT + TSS_sham_ group in either the more impaired (**upper left**) or less impaired (**lower left**) limb. Likewise, no significant change in mean first swing excursion (FSE) was observed in participants in the LT + TSS group in either the more impaired (**upper right**) or less impaired (**lower right**) limb.

**Figure 7:**
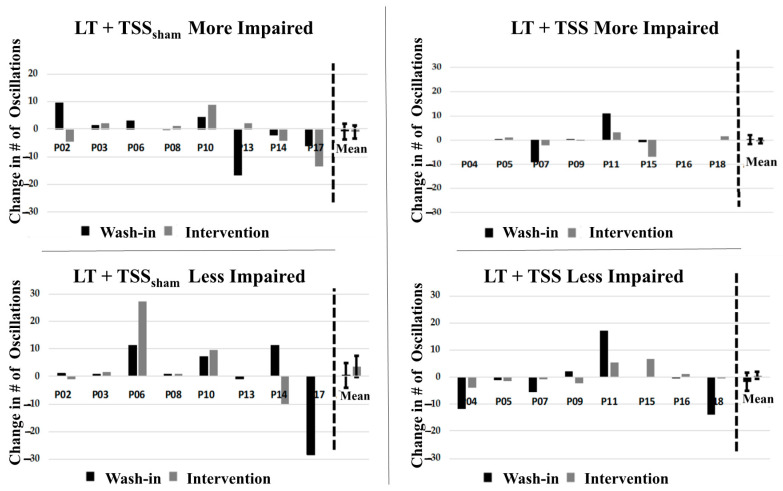
Drop test outcomes. No significant change in mean number of oscillations was observed in participants in the LT + TSS_sham_ group in either the more impaired (**upper left**) or less impaired (**lower left**) limb. Likewise, no significant change in mean number of oscillations was observed in participants in the LT + TSS group in either the more impaired (**upper right**) or less impaired (**lower right**) limb.

Spasticity outcome measures were not different at baseline between groups (Table 2). Our findings did not demonstrate significant changes over the course of the study for either quadriceps spasticity (Figure 6) or ankle clonus (Figure 7). Within-group comparisons yielded similar findings before and after training for both the LT + TSS and LT + TSS_sham_ groups for all the measures of interest (Table 2). Further, no significant differences between or within groups were found when SCATS was used to determine the immediate effects of TSS on spasticity.

Finally, at the beginning of the study, participants reported their spasticity to be mildly problematic, as determined by mSCI-SET (LT + TSS group: −0.22 (0.55); LT + TSS_sham_ group: −0.58 (0.31)). The mSCI-SET scores did not significantly change throughout the study for either of the two groups (wash-in phase: LT + TSS group: −0.31 (0.55); LT + TSS_sham_ group: −0.43 (0.18)/intervention phase: LT + TSS group: −0.24 (0.50); LT + TSS_sham_ group: −0.44 (0.13)). Effect sizes for all spasticity measures were small to medium.

## 4. Discussion

### 4.1. Feasibility

#### Integration into Clinical Practice

This study utilized a pragmatic clinical trial design to assess whether it is feasible to incorporate TSS into a clinical LT program and to acquire a preliminary evaluation of the efficacy of this combined training. The interest and willingness of participants to enroll in a study provides insight into the feasibility of a novel neuromodulation intervention. With a 17% enrollment rate, there was moderate interest in using TSS in conjunction with LT among those persons with subacute SCI. While this enrollment rate is relatively low compared with the number of potential participants (*n* = 107), it is higher than reported for other randomized LT studies enrolling participants during the subacute rehabilitation phase post-SCI [41] and in an exoskeleton LT study [42], wherein the enrollment rates were 9% and 12%, respectively. Moreover, a similar 17% enrollment rate was found in another exoskeleton study [43], indicating that there is a comparable interest among those with subacute SCI to try novel approaches to LT that have minimal risk. 

The interest in using TSS as an adjunct to LT may be higher than reported by the enrollment rate. At the time of this study, additional clinical trials were enrolling individuals with SCI from both in-patient and out-patient programs. While individuals often expressed interest in participating in all of the available studies, unknown carryover effects prevented individuals from simultaneously participating in multiple studies. Interestingly, when persons with SCI met criteria for both studies and had to choose between the current study of improving walking function and lower extremity spasticity versus a study focused on neuromodulation for improving hand function, 8 individuals declined to participate in the current study and chose the study focused on hand function. This observation is similar to findings in the literature, wherein when asked to rank functional priorities that would most enhance their quality of life, individuals with tetraplegia ranked improving hand function higher than improving walking function [44]. 

In assessing study feasibility, we identified early study design obstacles that slowed participant recruitment and that may have also impacted the overall enrollment rate. The design did not fully anticipate the concerns of participants with subacute SCI enrolled in a daily, intensive rehabilitation program with the time required for multiple weekly research assessments. To address this concern, assessment time points were reduced from twice per week (i.e., at the start and end of each week) to once per week (i.e., at the start and end of the 2-week wash-in period, and at the start and end of the 2-week intervention period). It should be noted that these concerns were due to the study assessments and not to the application of TSS as an adjunct intervention. For a typical 1 h LT session (i.e., usual care) with setup time, patients usually walk for approximately 30 min. No difference was found in LT walking time between wash-in and intervention weeks, indicating that TSS can be applied as an adjunct without significant interference with LT. An additional design issue that may have impacted the enrollment rate was recruiting only from patients enrolled in the Shepherd Center’s day program. The duration of services for the day program is based on therapy goals and progress, and therefore is individualized for each patient. For some patients, this time may be less than 4 weeks, making them ineligible for the study and ultimately slowing recruitment. To facilitate enrollment, we revised the study protocol to include individuals participating in the in-patient locomotor training program, which utilized an identical clinician-driven LT program, transitioned into the day program LT, and allowed for 4 weeks of LT. Collectively, these design issues may have negatively impacted the enrollment rate, skewing the perception of interest in using TSS as an adjunct LT intervention.

### 4.2. Safety and Tolerability

The application of TSS was found to be safe and tolerable, with no study-related adverse events being reported. Of the 18 participants enrolled in the study, 16 (89%) completed the study. The high completion rate of this study is comparable to those reported in other LT feasibility studies in persons with subacute [45] and chronic SCI [46], and falls within the studies anticipated 15% attrition rate. TSS was found to be generally tolerable with some reported discomfort and a pain rating of 4 out of 10. Participants often described feeling intense tightness in the abdomen and lower back around the site of electrodes. 

While the stimulation intensity was set at a level below motor threshold for the legs and at a level that produced paresthesia in the legs, the stimulation intensity would activate paraspinal muscles and abdominal muscles, as observed from visual inspection, and which may have contributed to the experienced discomfort. The contraction of abdominal and paraspinal muscles is commonly reported in other TSS studies [5,25]; however, the tolerability of the stimulation appears to vary based on how TSS is applied. In short bursts, TSS was not found to cause discomfort [5,25]. In a case series of persons with chronic SCI undergoing LT combined with TSS, TSS was found to be minimally painful with a mean numeric rating scale for pain of 0.12 and a range in responses from 0 to 4 out of 10 [12]. It is possible that the larger rectangular electrode used in the case series helped to disperse the sensation of stimulation on the lower back, providing a different mean pain rating than observed in this current study. Nonetheless, while TSS may be uncomfortable for some individuals, the sensation of TSS is within a tolerable pain range that did not interfere with LT. These findings indicate that TSS can be a feasible adjunct treatment for LT. 

In recent years, there has been growing interest in the use of neuromodulation approaches to promote use-dependent plasticity and augment the effects of LT on walking function. Epidural stimulation studies focus on improving walking function in persons with severe SCI, but they often consist of extensive LT schedules with over 250 training sessions [47] that are unrealistic to incorporate into usual clinical care. TSS studies have shown promise in improving walking function but also lack a control group to accurately assess efficacy [5,12]. We specifically designed this study to be integrated into standard clinical care consisting of 1 h LT sessions, 3 days a week, with an appropriate control group to better assess the preliminary efficacy of TSS on three aspects of walking function: walking speed, distance (endurance), and symmetry. 

### 4.3. Preliminary Efficacy—Walking Function

During the 2-week wash-in phase, wherein all participants received LT alone, the change in walking speed was significant for both groups. The LT + TSS group (0.16 m/s) exceeded the threshold of 0.13 m/s for clinically relevant change [48], while the change for the LT + TSS_sham_ group (0.12 m/s) was within 0.01 m/s of the threshold (see Table 2). Further, both groups exhibited large effect sizes for walking speed during the wash-in phase. The finding that there was meaningful improvement in speed during the first 2 weeks suggests that there is a rapid improvement in walking speed early after the onset of LT. As has been noted in the literature, when individuals are exposed to new activities, it is not unusual to observe early improvements regardless of the intervention, and this phenomenon is the rationale for recommending use of a wash-in (phase-in) study design [49] such as that we used in our study. 

Following the wash-in phase, during the subsequent 2-week intervention phase, walking speed in the LT + TSS group also improved by 0.16 m/s, continuing to meet the threshold for clinically relevant change, and this change was statistically significant with a large effect size. Conversely, in the LT + TSS_sham_ group, performance plateaued during the wash-in phase, such that the change of 0.06 m/s during the intervention phase failed to meet the threshold for clinically relevant change and was not significant. The inability to detect a significant difference between the LT + TSS and LT + TSS_sham_ groups during the intervention phase may have been due to the failure to meet the recruitment target of 24 participants on which the study was powered. 

As with walking speed, the changes in 2MWT distance were similar during the LT-only wash-in phase for the LT + TSS and the LT + TSS_sham_ groups, at 16.58 and 19.08 m, respectively. This change was not significant for the LT + TSS group but was significant for the LT + TSS_sham_ group. However, the effect size was large in both groups. During the intervention phase, the LT + TSS group continued to improve with a change of 15.25 m, which was statistically significant with a large effect size. The change in the LT + TSS_sham_ group of 6.85 m was more modest, and not significant. Although threshold values for clinically relevant change in the 2MWT have not been established for persons with SCI, extrapolation from the 6MWT threshold of 45.8 m [48] would suggest that a change of 16.3 m is a clinically relevant change. The change in the 2MWT distance in the LT + TSS group during the intervention phase fell short of this threshold by 1 m. 

### 4.4. Preliminary Efficacy—Spasticity

Prior studies of locomotor training in persons with SCI have demonstrated that walking is associated with reductions in spasticity [21,22,23,28], and growing evidence suggests that TSS can reduce spasticity as well [24,25,28]. For this reason, the finding of no significant between-group or within-group effects in our clinical measure of immediate post-treatment effects (SCATS) or our measures of persistent (one day after intervention) effects on quadriceps spasticity (pendulum test) or ankle plantar flexor clonus was unexpected. The literature indicates that an FSE of 96 degrees represents the 95th percentile for minimum FSE in individuals without neurologic conditions [29]. Based on our FSE data, there are some individuals who approached this value, indicating minimal excitability of the quadriceps stretch reflex. Conversely, in other participants, the small FSE angle indicates greater excitability of this reflex. Likewise, prior work has indicated that individuals exhibiting 4 or more oscillations of clonic activity following biomechanical stretch of the plantar flexors also exhibit electrophysiologic evidence of impaired reflex modulation [50]. In the TSS group, the number of clonic oscillations fell within the range of neurologically intact individuals in both the more- and less-impaired legs. However, the TSS_sham_ group demonstrated greater levels of clonic activity in both legs. It is possible that the reason we failed to identify an effect of TSS on spasticity is that the pendulum test and drop test assessments were performed the day following the last intervention. In one prior study of TSS, the antispasmodic effects persisted for up to 7 days, although inspection of the published graphical data indicates that the pendulum test values were highly variable over the course of that study [24]. It may be that SCATS, while a fast, low-tech clinical tool to assess severity of multiple manifestations of spasticity, is not sufficiently sensitive for determining small changes in spasticity, as an ordinal measure. Nonetheless, while the lack of effects on spasticity in the present study were unexpected and may be due to study design limitations, the effects of TSS and LT on spasticity are unclear and warrant further study to understand its efficacy as an approach for management of spasticity. 

### 4.5. Limitations

All participants were in the subacute stage of injury, a time that is thought to have the greatest opportunity for neural plasticity [51]. The fact that this early post-injury period is the time when large changes in function are most likely means that it is also a phase of injury wherein there is a large amount of inherent variability in the course of recovery. The effects of inter-individual differences in patterns of recovery may have been compounded by variability in the rehabilitation program among the participants. For most participants, the LT sessions consisted of both overground and treadmill-based training sessions, with the amount of each dependent on clinical judgement of the treating physical therapist. While prior work has shown that outcomes are equivalent for these two LT approaches when used in the subacute stage of SCI [41], differences in the type of LT received, as well as variations in the types and amounts of other forms of physical therapy, could have added to the variability in outcomes, making it more difficult to discern treatment effects. 

The pragmatic nature of our study design required that set-up time be maintained at a minimum with an emphasis on approaches that can be readily incorporated into clinical practice. A limitation of this approach is that we used observation of visible muscle contraction to verify that the stimulation was subthreshold for a motor response rather than measuring electromyographic output. For this reason, it is possible that the stimulation intensity was not optimal for achieving activation of the target neural structures.

The slow initial enrollment rate and withdrawal of two subjects left the study underpowered to detect between-group differences. Likewise, the lack of significant within-group changes in the LT + TSS_sham_ group may also reflect the small sample size and highlights that efficacy findings should be interpreted cautiously. Future studies sufficiently powered to assess these differences will provide better insight into the efficacy of TSS for augmenting the effects of LT. 

## 5. Conclusions

Combination approaches for improving walking function in persons with SCI have been cited most likely to result in meaningful change [52]. Here, we demonstrated that applying TSS as an adjunct to LT is both feasible and tolerable when applied as part of the usual care in a clinical setting. Moreover, the improvements in walking outcomes provides insight into the potential benefits of this combined approach for improving walking function and warrant further research aimed at understanding the optimal stimulation parameters for improving functional outcomes in persons with subacute as well as chronic SCI.

## Figures and Tables

**Table 1:** Demographics.

Subject ID	Sex	Age (Years)	Time Since Injury (Days)	AIS	Neurological Injury Level	LEMS (R)	LEMS (L)	LEMS(Total)
1 *	M	51	56	D	C4	20	17	37
2	M	43	80	C	C4	14	13	27
3^†^	M	65	88	D	C5	20	22	42
4	F	53	36	D	C2	22	20	42
5	M	56	84	D	C4	20	24	44
6	M	37	103	C	C3	17	22	39
7 ^†,‡^	F	55	75	C	C3	9	11	20
8	M	47	119	D	C2	21	21	42
9 ^‡^	M	18	83	D	C7	22	11	33
10	F	24	82	C	C7	17	21	38
11 ^†,‡^	M	32	121	C	C4	24	6	30
12 *	M	54	171	C	C4	14	22	36
13	M	20	68	D	C4	11	25	36
14	M	52	195	B	T11	22	19	41
15 ^‡^	M	54	141	D	C5	18	14	32
16	M	63	185	D	C1	22	24	46
17 ^†^	F	58	71	D	C6	8	22	30
18	M	18	47	D	C5	25	8	33

Abbreviations: AIS, American Spinal injury Association Impairment Scale (C: Motor function is preserved below the neurological level, and more than half of key muscles below the neurological level have a muscle grade less than 3; D: Motor function is preserved below the neurological level, and at least half of key muscles below the neurological level have a muscle grade of 3 or more); LEMS, Lower Extremity Motor Score. * participants who withdrew from the study; † participants with insufficient walking ability to complete walking tests; ‡ participants in the locomotor training and transcutaneous spinal cord stimulation (LT + TSS) group who reported tingling in the legs during stimulation.

**Table 2:** Walking and spasticity outcomes.

		Wash-in Phase					Intervention Phase			
	T1	T2	Difference	*p*-Value	Effect Size	T3	T4	Difference	*p*-Value	Effect Size
**Walking Assessments**										
Walking Speed (m/s)										
LT + TSS	0.47 (0.25)	0.63 (0.30)	0.16 (0.11)	0.05	0.53	0.66 (0.38)	0.82 (0.28)	0.16 (0.13)	0.03	0.43
LT + TSS_sham_	0.18 (0.08)	0.30 (0.11)	0.12 (0.05)	0.03	1.15	0.32 (0.14)	0.38 (0.21)	0.06 (0.08)	0.12	0.31
Walking Distance (m)										
LT + TSS	57.07 (7.88)	73.65 (27.64)	16.58 (20.21)	0.12	0.66	69.27 (28.84)	84.52 (29.30)	15.25 (9.81)	0.03	0.48
LT + TSS_sham_	22.70 (2.81)	39.13 (14.69)	19.08 (13.17)	0.04	1.35	45.05 (18.43)	51.90 (23.59)	6.85 (7.82)	0.12	0.30
Step Length Asymmetry (unitless)										
LT + TSS	0.54 (0.02)	0.54 (0.02)	0.00 (0.03)	0.92	0.09	0.53 (0.01)	0.53 (0.02)	0.00 (0.02)	0.75	0.13
LT + TSS_sham_	0.57 (0.07)	0.54 (0.05)	−0.03 (0.06)	0.27	0.49	0.54 (0.03)	0.52 (0.02)	−0.02 (0.03)	0.47	0.68
**Spasticity Assessments**										
Pendulum: More Impaired LE (degrees)										
LT + TSS	60.00 (11.84)	54.61 (21.95)	−5.39 (22.32)	0.40	0.29	54.18 (20.67)	54.90 (15.22)	0.72 (8.88)	0.89	0.04
LT + TSS_sham_	60.43 (23.60)	63.24 (18.83)	2.81 (19.00)	0.48	0.12	57.42 (19.74)	63.98 (20.04)	6.56 (18.00)	0.40	0.31
Pendulum: Less Impaired LE (degrees)										
LT + TSS	67.42 (20.06)	69.47 (24.98)	2.05 (16.09)	0.48	0.09	64.97 (34.24)	71.55 (27.90)	6.58 (13.19)	0.26	0.20
LT + TSS_sham_	60.52 (22.06)	66.62 (19.01)	6.10 (15.88)	0.26	0.28	56.72 (16.86)	63.61 (22.19)	6.88 (18.72)	0.16	0.33
Ankle clonus: More Impaired LE (number of oscillations)										
LT + TSS	8.21 (7.10)	6.54 (7.58)	−1.67 (9.62)	0.50	0.21	9.88 (10.03)	10.42 (11.57)	0.54 (3.69)	1.00	0.05
LT + TSS_sham_	14.75 (16.36)	13.96 (16.63)	−0.79 (7.93)	1.00	0.05	14.13 (15.83)	13.19 (18.51)	−0.94 (6.57)	0.74	0.05
Ankle clonus: Less Impaired LE (number of oscillations)										
LT + TSS	4.67 (2.87)	4.83 (4.86)	0.17 (5.47)	0.89	0.04	6.29 (6.62)	5.88 (7.52)	−0.42 (3.08)	0.92	0.06
LT + TSS_sham_	17.83 (19.04)	18.25 (22.48)	0.42 (12.62)	0.23	0.02	12.38 (18.47)	16.00 (21.97)	3.63 (10.96)	0.35	0.17

Abbreviations: T1, testing session 1; T2, testing session 2; T3, testing session 3; T4, testing session 4; LT + TSS, locomotor training and transcutaneous spinal cord stimulation group; LT + TSS_sham_, locomotor training and sham stimulation group; LE, lower extremity.

## Data Availability

The data presented in this study are available upon request from the corresponding author.
